# A Novel Antimicrobial–Phytochemical Conjugate With Antimicrobial Activity Against *Streptococcus uberis*, *Enterococcus faecium*, and *Enterococcus faecalis*

**DOI:** 10.3389/fphar.2019.01405

**Published:** 2019-11-28

**Authors:** Karthic Rajamanickam, Jian Yang, Meena Kishore Sakharkar

**Affiliations:** Drug Discovery and Development Research Group, College of Pharmacy and Nutrition, University of Saskatchewan, Saskatoon, SK, Canada

**Keywords:** antimicrobial resistance, antimicrobial–phytochemical conjugate, chemical synthesis, minimum inhibitory concentration, *Streptococcus*, *Enterococcus*

## Abstract

Antimicrobial resistance is one of the major threats to human and animal health. An effective strategy to reduce and/or delay antimicrobial resistance is to use combination therapies. Research in our laboratory has been focused on combination therapies of antimicrobials and phytochemicals and development of antimicrobial–phytochemical conjugates. In this study, we report the synthesis and antimicrobial activity of a novel sulfamethoxazole–gallic acid conjugate compound (Hybrid 1). Hybrid 1 not only showed much stronger activity than sulfamethoxazole towards *Streptococcus uberis* 19436, *Enterococcus faecium* 700221, and *Enterococcus faecalis* 29212, which were purchased from American Type Culture Collection (ATCC), but also exhibited a promising antimicrobial effect against two *E. faecalis* clinical isolates, one of which was multidrug-resistant. Further studies are warranted to establish the *in vivo* antimicrobial activity for Hybrid 1 and develop more potent sulfamethoxazole–gallic acid-based antimicrobial conjugates using hybrid 1 as a lead compound.

## Introduction

Antimicrobialresistance, a key cause of morbidity and mortality, has been emerging as one of the main threats to human and animal health (Laxminarayan et al., 2013; Laxminarayan et al., 2016). Overconsumption and misuse of antimicrobials is the primary driver of antimicrobial resistance (Steinke and Davey, 2001; Goossens et al., 2005; Malhotra-Kumar et al., 2007). Recently, Klein et al. reported the antibiotic consumption in 76 countries between 2010 and 2015 and projected a future increase in global antibiotic consumption (Klein et al., 2018). Furthermore, overuse and/or improper use of antimicrobials has caused a significant increase of multidrug-resistant microbes, which have become an urgent issue facing medical sciences and might even impose a potential pandemic catastrophe (The Lancet, 2014; World Health Organization, 2018). Therefore, reducing usage and dosage is essential in preventing the development of antimicrobial resistance.

*Streptococcus* spp., which are usually divided into α-hemolytic streptococci and β-hemolytic streptococci, are a genus of Gram-positive cocci. They are among the most frequent cause of infections in human and animals (Patterson, 1996). *Enterococcus*spp., which are formerly known as group D of streptococci, were classified as a different genus in 1984 (Schleifer and Kilpper-Bälz, 1984). They can be found everywhere, including the intestines and feces of birds, animals, and human, and are important pathogens. For example, *Enterococcus faecalis*is one of the major bacteria in hospital-acquired infection (HAI), bovine mastitis, and enterococcosis in poultry. Both streptococci (Haenni et al., 2018) and enterococci (Miller et al., 2014) develop antimicrobial resistance rapidly, which, in turn, not only impose a serious risk to human and animal health but also cause a huge economic loss. Although antimicrobial combination therapies are normally used to reduce antimicrobial resistance, many bacteria have developed resistance to such therapies (i.e., multidrug resistance) (Nikaido, 2009; Laxminarayan et al., 2013).

Our previous studies have shown that some phytochemicals possess antimicrobial activities and exhibit synergistic/additive effects with antimicrobials (Jayaraman et al., 2010; Jayaraman et al., 2011; Rajamanickam et al., 2019a; Rajamanickam et al., 2019b). Therefore, co-administration of phytochemicals can significantly reduce the dosage and usage of antimicrobials. Phytochemicals are naturally occurring secondary metabolites in plants. They are relatively safe to use and do not leave toxic residues. However, the biodistribution and metabolism profiles of the phytochemicals may not coincide with those of the antimicrobials, and thus challenging the antimicrobial–phytochemical combination therapies on whether the optimal therapeutic efficacy is really achieved. To overcome this obstacle, we adopted an *in silico* approach to design novel antimicrobial–phytochemical conjugate compounds (i.e., conjugate the active parts of antimicrobials and phytochemicals based on computer-aided molecular simulations) (Jayaraman et al., 2013). In the current study, we report the synthesis of a sulfamethoxazole–gallic acid conjugate (hybrid 1) and the evaluation of its antimicrobial activity against three bacterial stains purchased from the American Type Culture Collection (ATCC) and two*E. faecalis* clinical isolates (designated as isolates 1 and 2) from a Saskatchewan poultry farm. The three ATCC strains are *Streptococcus uberis* 19436, *Enterococcus faecium* 700221, and *E. faecalis* 29212, and the *E. faecalis* clinical isolate 2 was a multidrug-resistant strain.

## Methods

### Bacterial Strains, Culture Media, and Chemicals

*S. uberis* 19436, *E. faecium* 700221, and *E. faecalis* 29212 were purchased from ATCC (Manassas, VA, USA). *E. faecalis* clinical isolate 1 and *E. faecalis* clinical isolate 2 (multidrug-resistant) were collected from a Saskatchewan poultry farm. Cell culture media for these bacterial strains were purchased from Cedarlane Canada (Burlington, ON, Canada). Gallic acid was purchased from ThermoFisher Scientific (Ottawa, ON, Canada). Sulfamethoxazole and other chemicals were purchased from Sigma-Aldrich Canada (Oakville, ON, Canada).

### Synthesis of Compound Hybrid 1

**Figure 1:**
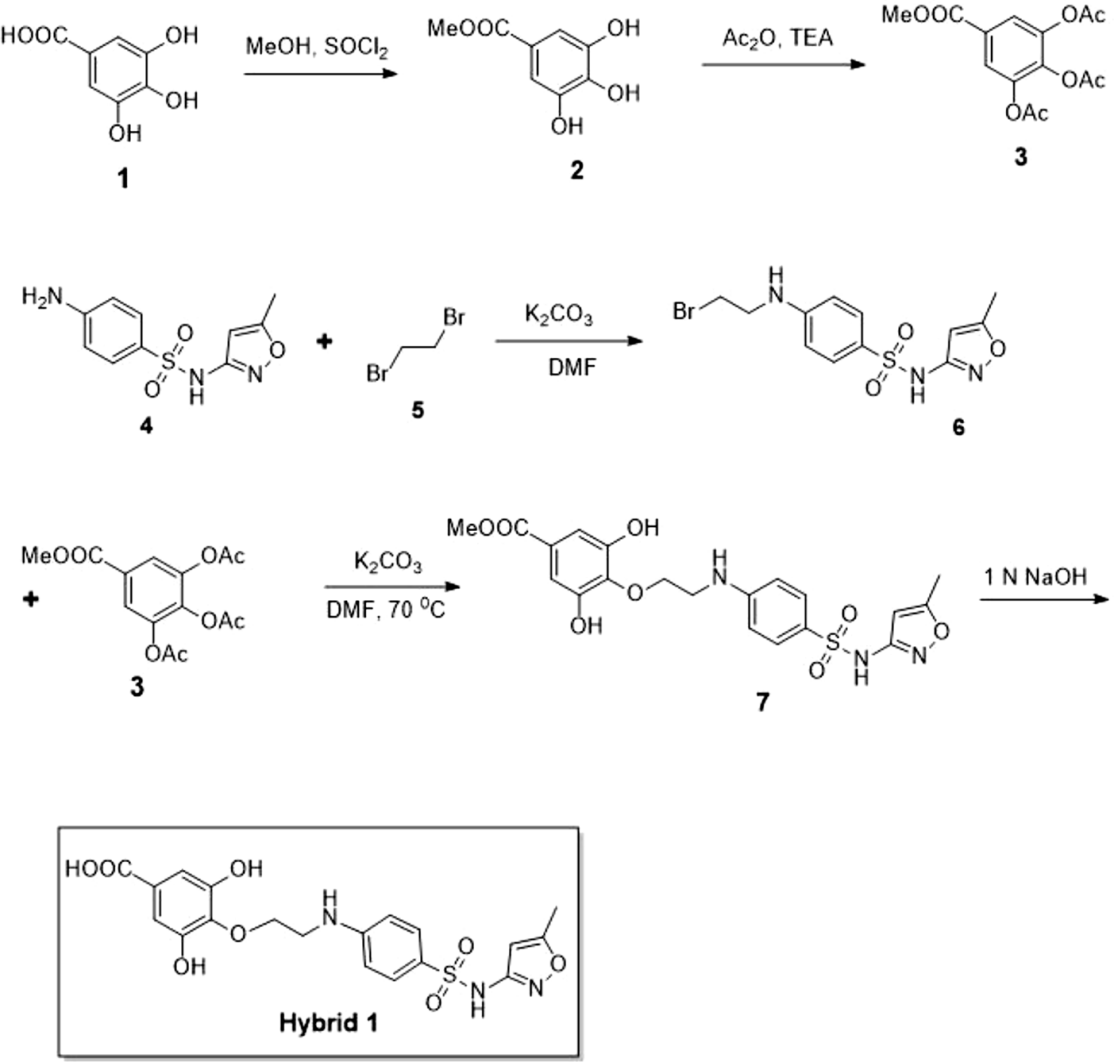
Synthesis procedure of the novel sulfamethoxazole–gallic acid conjugate Hybrid 1.

Synthesis procedure of compound Hybrid 1 is illustrated in **Figure 1**. Compound 2 was synthesized by adding thionyl chloride (4.19 g, 35.29 mmol) into a suspension solution of gallic acid (compound 1, 5 g, 29.41 mmol dissolved in 50 ml methanol) at 0°C. The reaction mixture was under stirring at room temperature for 5 h. Upon completion of the reaction, compound 2 was obtained as a white solid by vacuum evaporation and drying. Compound 3 was synthesized by adding triethylamine (6.6 g, 65.22 mmol) and acetic anhydride (6.5 g, 63.67 mmol) into a suspension solution of compound 2 (2 g, 21.72 mmol dissolved in 30 ml dichloromethane) at 0°C. The reaction mixture was under stirring at room temperature for 2 h. Upon completion of the reaction (confirmed by thin-layer chromatography), the reaction mixture was diluted with water and extracted by dichloromethane thrice. The collected organic phase was dried over anhydrous Na_2_SO_4_, concentrated, and purified by column chromatography (12% ethyl acetate in hexane) to obtain compound 3 as a white solid (5.0 g, 16.12 mmol, 74% yield). The structure of compound 3 was confirmed by^1^H NMR (500 MHz, CDCl3): δ 7.80 (s, 2H), 3.90 (s, 3H), 2.30 (s, 3H), 2.29 (s, 3H), and LC-MS (electrospray ionization, ESI): [M+H]^+^, 332.96. Compound 6 was synthesized by adding dibromoethane (compound 5, 741 mg, 3.94 mmol) and potassium carbonate (682 mg, 4.94 mmol) into a solution of sulfamethoxazole (compound 5, 500 mg, 1.97 mmol) in 12 ml dimethylformamide at 0°C. The reaction mixture was under stirring at room temperature for 1 h. Upon completion of the reaction (confirmed by thin-layer chromatography), the reaction mixture was extracted with ethyl acetate twice. The collected organic phase was dried over anhydrous Na_2_SO_4_, concentrated, and purified by column chromatography (9% ethyl acetate in hexane) to obtain compound 6 as a colorless oil (450 mg, 1.25 mmol, 63% yield). LC-MS (ESI) identified [M+H]^+^of 360.01. Compound 7 was synthesized by slowly adding potassium carbonate (230 mg, 1.66 mmol) into a solution of compounds 6 (300 mg, 0.83 mmol) and 3 (284 mg, 0.91 mmol) in 10 ml dimethylformamide at 0°C. The reaction mixture was under stirring at room temperature for 1 h and subsequently at 70°C for 4 h. Upon completion of the reaction, the reaction mixture was extracted with ethyl acetate twice. The collected organic phase was dried over anhydrous Na_2_SO_4_, concentrated, and purified by column chromatography (60% ethyl acetate in hexane) to obtain compound 7 as a colorless oil (100 mg, 0.21 mmol, 26% yield). LC-MS (ESI) identified [M+H]^+^of 464.04. Finally, compound Hybrid 1 was synthesized by slowly adding 1.0 M NaOH solution into a solution of compound 7 (90 mg, 0.19 mmol) in 10 ml NaOH (1.0 M) under stirring at 0°C. The reaction mixture was continuously under stirring at room temperature for 0.5 h with reaction progress monitored by thin-layer chromatography. Upon completion of the reaction, the reaction mixture was extracted with ethyl acetate twice. The collected aqueous phase was neutralized with sodium hydrogen sulfate and then extracted with ethyl acetate twice. The organic phase was dried over anhydrous Na_2_SO_4_, concentrated, and purified by column chromatography (80% ethyl acetate in hexane) to obtain compound Hybrid 1 as a white solid (70 mg, 0.15 mmol, 82% yield, purity of 98.4% based on LC-MS).

### Determination of MIC

In this study, minimum inhibitory concentration (MIC) refers to the lowest concentration of an antimicrobial agent (Hybrid 1 or sulfamethoxazole) to inhibit the visible growth of a microorganism (equivalent to ∼85% growth inhibition based on OD_655_ measurement using a Bio-Rad iMark Microplate Reader) after 18–24 h incubation. The protocol used to determine MIC of Hybrid 1 towards the bacterial stains has been published previously (Rajamanickam et al., 2019a). The concentration of Hybrid 1 ranges between 9.38 and 1,200 µg/ml towards *S. uberis* 19436, *E. faecium* 700221, and *E. faecalis* 29212 using sulfamethoxazole as a control (concentration range, 9.38–1,200 µg/ml), whereas the concentration of Hybrid 1 ranges between 15.62 and 2,000 µg/ml towards the two *E. faecalis* clinical isolates. The treatment time was 18–24 h.

### Statistical Analysis

All experiments were performed in triplicate and statistical analyses were performed using GraphPad Prism 5.0 statistical software (GraphPad Software, La Jolla, CA, USA). The experimental data were analyzed by one-way ANOVA with *post hoc* Tukey’s multiple comparison test with significance set at *p* ≤ 0.05 (*p ≤ 0.05, **p ≤ 0.01). Correlation coefficient (*r*) value was calculated by using Pearson’s correlation method.

## Results and Discussion

**Figure 2:**
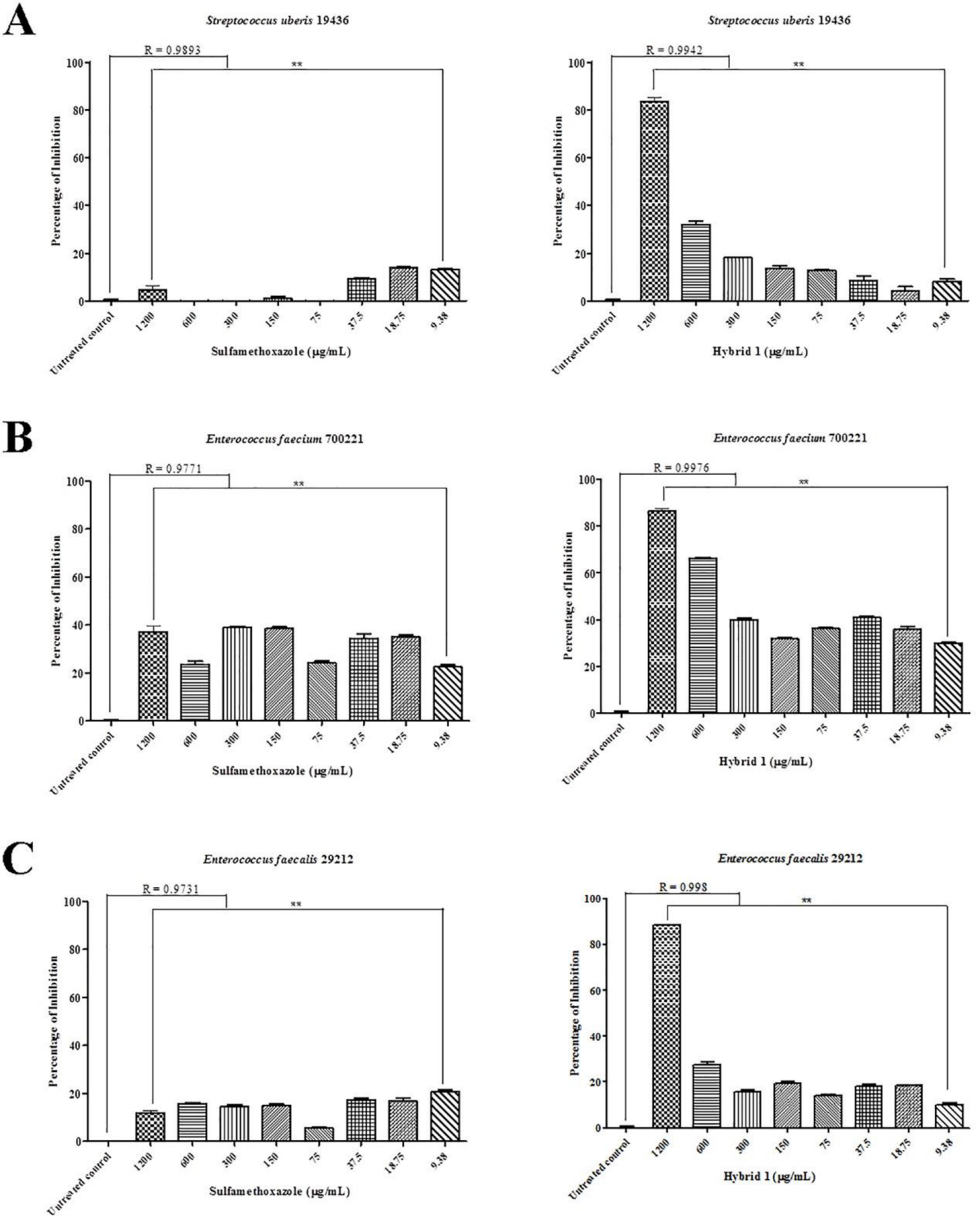
Antimicrobial activity of sulfamethoxazole and Hybrid 1 towards *Streptococcus uberis* 19436 **(A)**, *Enterococcus faecium*700221 **(B)**, and *Enterococcus faecalis* 29212 **(C)** using a protocol developed in our laboratory (Rajamanickam et al., 2019a). The concentrations of both sulfamethoxazole and Hybrid 1 range from 9.38 to 1,200 µg/ml. The experiment was carried out in triplicate and the data were analyzed with one-way ANOVA with *post hoc* Tukey’s multiple comparison test using GraphPad Prism 5 (GraphPad Software, La Jolla, CA, USA). The significance was set at *p* ≤ 0.05 (*p ≤ 0.05, **p ≤ 0.01).

**Figure 3:**
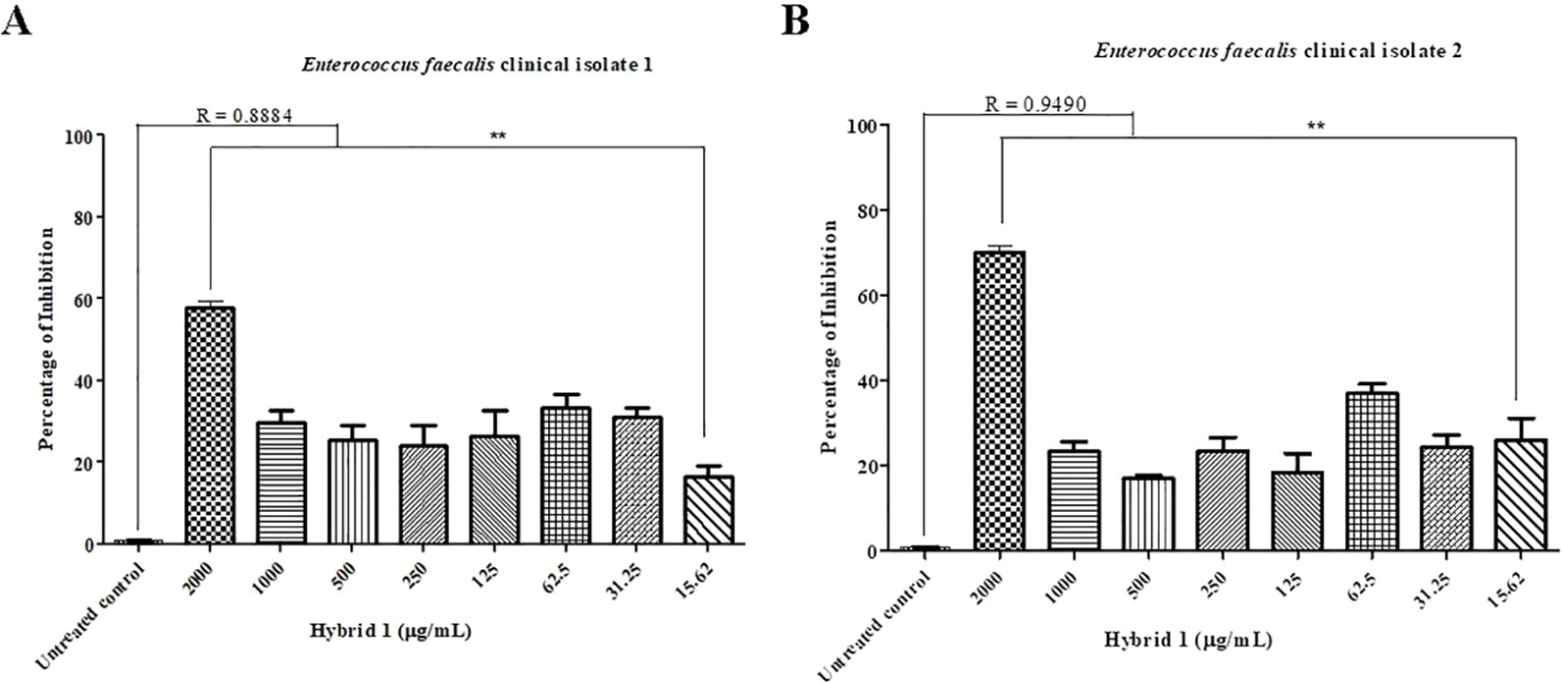
Antimicrobial activity of Hybrid 1 towards *Enterococcus faecalis* clinical isolate 1 **(A)** and multidrug-resistant *Enterococcus faecalis* clinical isolate 2 **(B)**, which were isolated from a Saskatchewan poultry farm. The concentration of Hybrid 1 ranges from 15.62 to 2,000 µg/ml. The experiment was carried out in triplicate and the data were analyzed by one-way ANOVA with*post hoc*Tukey’s multiple comparison test using GraphPad Prism 5 (GraphPad Software, La Jolla, CA, USA). The significance was set at *p* ≤ 0.05 (*p ≤ 0.05, **p ≤ 0.01).

Synthesis procedure of the novel sulfamethoxazole–gallic acid conjugate Hybrid 1 is shown in **Figure 1**. The chemical structure of Hybrid 1 was characterized by^1^H NMR (500 MHz, CDCl3): δ 7.44 (2H, d, *J*= 14.5 Hz), 6.92 (2H, s), 6.59 (2H, d, *J*= 15.0 Hz), 6.39 (1H, s), 6.23 (2H, s), 4.15–4.11 (2H, m), 4.06–3.97 (2H, s), 2.34 (3H, s), and LC-MS (ESI): [M+H]^+^, 450.49 (**Supplementary Figure S1**). The antimicrobial activity of compound Hybrid 1, with sulfamethoxazole as a control, was firstly evaluated towards the three ATCC strains— *S. uberis* 19436, *E. faecium* 700221, and *E. faecalis* 29212—using a protocol developed in our laboratory (Rajamanickam et al., 2019a). *S. uberis* is the most common bacterial mastitis-causing pathogen in lactating cows worldwide (Leigh, 1999; Valentiny et al., 2015), although *Staphylococcus aureus* is probably the most common pathogenic bacterium in Canadian dairy farms (Olde Riekerink et al., 2008). As shown in **Figure 2A**, sulfamethoxazole did not inhibit the growth of *S. uberis* 19436, which is consistent with previous reports that *S. uberis* isolates from dairy cows with mastitis are highly resistant to sulfamethoxazole (Phuektes et al., 2001; McDougall et al., 2014). However, *S. uberis* 19436 was responding to Hybrid 1, with the MIC of Hybrid 1 measured at 1,200 µg/ml. *E. faecium* and *E. faecalis* are the most common *Enterococcus* spp. in not only hospital-acquired infections but also enterococcal infections on dairy and poultry farms. For example, Tyson et al. recently assessed the prevalence and antimicrobial resistance of enterococci isolated from retail meats in the United States between 2002 and 2014 and found that >90% of meats are contaminated with enterococci (Tyson et al., 2017). Sulfamethoxazole gave a non-concentration-dependent inhibition of 25–40% on the growth of *E. faecium* 700221 and of 10–20% on the growth of *E. faecalis* 29212, respectively (**Figures 2B, C**). However, Hybrid 1 exhibited a much stronger antimicrobial activity than sulfamethoxazole, with the respective MIC of 1,200 µg/ml for both *Enterococcus* strains. Furthermore, we examined the antimicrobial activity of Hybrid 1 towards two *E. faecalis* clinical isolates collected from a Saskatchewan poultry farm (**Figure 3**). Hybrid 1 gave a ∼60% inhibition on the growth of *E. faecalis* clinical isolate 1 and a 70% inhibition on the growth of the multidrug-resistant *E. faecalis* clinical isolate 2. These promising *in vitro* results implicate that Hybrid 1 serves as a good lead compound to develop new conjugates (i.e., chemical analogues of Hybrid 1) which possess more potent antimicrobial activities, although the high MICs might limit its potential clinical usage. Further studies, such as mouse air pouch model, calf infection model, and pharmacokinetic evaluation, are warranted to establish the *in vivo* antimicrobial activity and structure–activity relationship (SAR) for Hybrid 1 and its chemical analogues.

## Conclusion

In this study, we designed and synthesized a novel antibiotic–phytochemical conjugate compound, Hybrid 1, and showed it has potent antimicrobial activity towards not only three ATCC strains—*S. uberis* 19436, *E. faecium* 700221, and *E. faecalis* 29212—but also two *E. faecalis* clinical isolates. The current study suggests that co-administration of antimicrobials and phytochemicals and development of antimicrobial–phytochemical conjugates may be a valid and promising strategy in tackling antimicrobial resistance in bacteria.

## Data Availability Statement

The datasets generated for this study are available on request to the corresponding authors.

## Author Contributions

This work was designed by JY and MS and carried out by KR. The manuscript was written by JY and MS and approved for publication by all authors.

## Conflict of Interest

The authors declare that the research was conducted in the absence of any commercial or financial relationships that could be construed as a potential conflict of interest.

## Supplementary Material

Supplementary Figure1 Mass spectroscopy of hybrid 1.
