# Supplementary figures and images for: A Novel Antimicrobial–Phytochemical Conjugate With Antimicrobial Activity Against Streptococcus uberis, Enterococcus faecium, and Enterococcus faecalis
Source: Front Pharmacol. 2019 Nov 28;10:1405. doi: 10.3389/fphar.2019.01405 (PMC6893902; doi:10.3389/fphar.2019.01405)

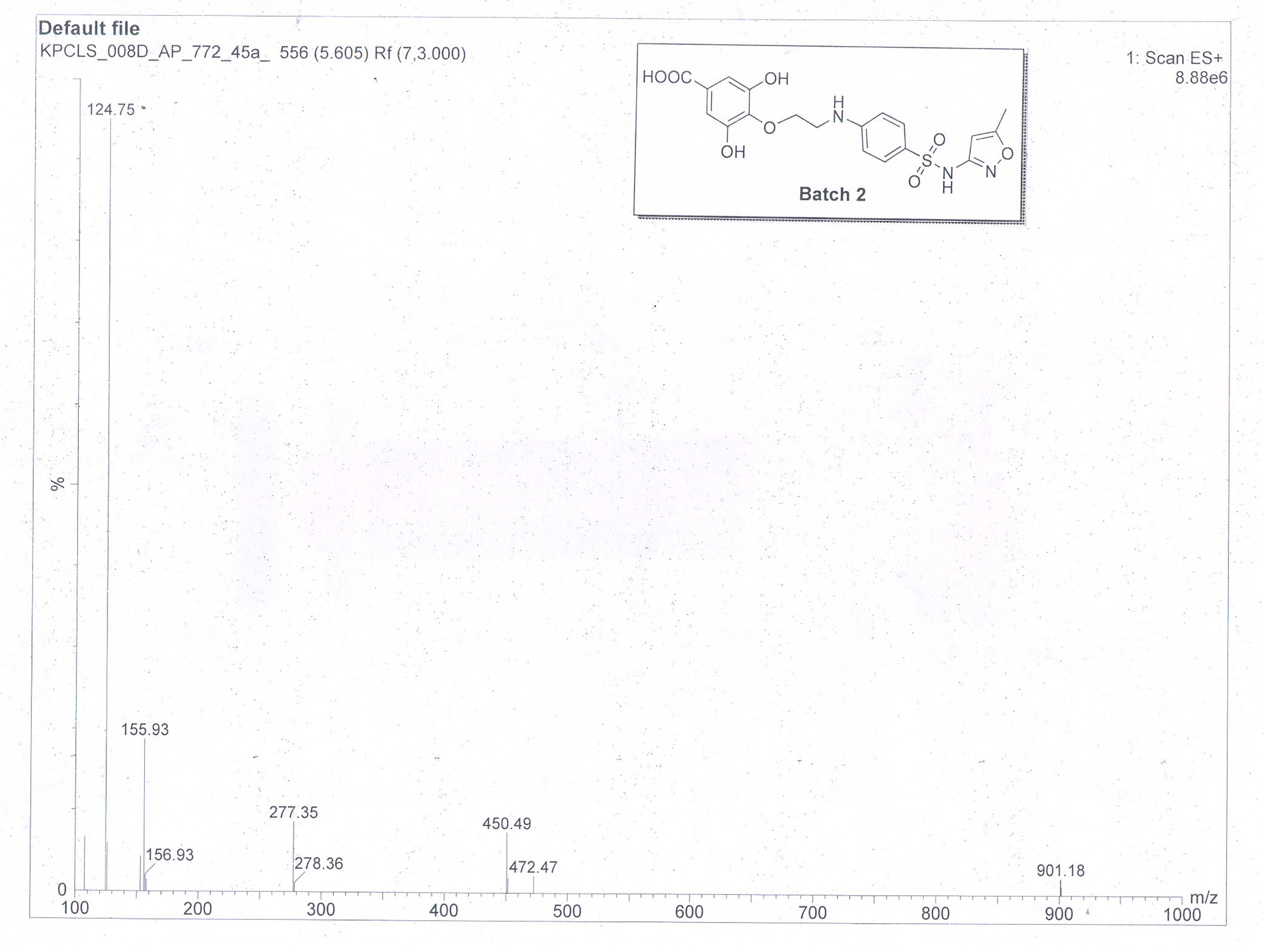

Supplement: Supplementary Figure1 — Mass spectroscopy of hybrid 1. [file Image_1.jpeg]
